# Angina in the young: a right coronary artery malignant course—a case report

**DOI:** 10.1093/ehjcr/ytag807

**Published:** 2026-11-02

**Authors:** Ayobami B Omodara, Kamran Dawood, Seena Darwin, Azhar Khokar, Amer Qureshi

**Affiliations:** Department of Cardiology, Leeds General Infirmary, Leeds Teaching Hospitals NHS Foundation Trust, Great George Street, Leeds LS1 3EX, UK; Department of Cardiology, Southend Hospital, Mid & South Essex University Teaching Hospitals NHS Foundation Trust, Prittlewell Chase, Essex SS0 0RY, UK; Department of Cardiology, Southend Hospital, Mid & South Essex University Teaching Hospitals NHS Foundation Trust, Prittlewell Chase, Essex SS0 0RY, UK; Department of Cardiology, Southend Hospital, Mid & South Essex University Teaching Hospitals NHS Foundation Trust, Prittlewell Chase, Essex SS0 0RY, UK; Department of Cardiology, Southend Hospital, Mid & South Essex University Teaching Hospitals NHS Foundation Trust, Prittlewell Chase, Essex SS0 0RY, UK

**Keywords:** Anomalous right coronary artery, Ischaemic heart disease, Congenital coronary artery anomaly, Computed tomography coronary angiogram (CTCA), Exercise stress test, Anginal chest pain in young adults, Case report

## Abstract

**Background:**

Coronary artery anomalies are rare congenital defects that often remain undetected due to their asymptomatic nature. However, in certain cases, they represent an important cause of sudden cardiac death, particularly among younger individuals with low to intermediate risk factors for coronary artery disease.

**Case summary:**

We report the case of a 35-year-old woman who presented to the cardiology outpatient clinic with intermittent typical anginal chest pain. Despite having a low-to-moderate risk profile for ischaemic heart disease, limited to well-controlled hypertension and hypercholesterolaemia, further evaluation with computed tomographic coronary angiography revealed an anomalous right coronary artery originating from the left coronary sinus with a malignant course. An exercise stress echocardiogram demonstrated ischaemic electrocardiographic changes in the absence of physical symptoms. Her cardiovascular risk factors were managed with medications; however, due to evidence of inducible ischaemia, she was referred to a tertiary adult congenital heart disease centre for ongoing surveillance and consideration of appropriately timed surgical intervention.

**Conclusion:**

Although rare, anomalous coronary artery disease may represent a significant cause of angina in younger patients. A thorough clinical history remains essential in identifying this condition and facilitating potentially life-saving management.

Learning points

- Anomalous RCA from the left coronary sinus can cause ischaemia and raise the risk of sudden cardiac death in young patients; imaging may help identify high-risk anatomy.
- Management depends on symptoms and anatomy: Low-risk asymptomatic patients can be monitored, while symptomatic or high-risk patients may require surgical correction.
- Maintain a high index of suspicion for non-atherosclerotic causes of ischaemia, especially in young patients with unconventional cardiovascular risk factors.

## Introduction

Among the various forms of congenital heart disease, anomalous origin of the coronary arteries represents a distinct subset owing to its rarity and the potential for unexpected, sudden, and catastrophic adverse outcomes, particularly in young individuals. Coronary angiography identifies anomalous coronary artery origins in up to 5% of cases. Within this cohort, the incidence of aberrant right coronary artery (RCA) from left sinus is 0.06–0.5%^1^ while approximately 0.15% exhibit the highest-risk pattern characterized by the left coronary artery (LCA) arising from the right coronary sinus.^2^

It is reported that anomalously originating coronary arteries account for roughly 15% of sudden cardiac deaths in athletes, attributed to multiple proposed mechanisms including torsion, exercise-induced compression of proximal arterial segments, vasospasm, or ventricular arrhythmias secondary to myocardial scarring.^3^ According to Angelini, a popular classification system proposed by him categorizes anomalies based on anatomical features including ostial abnormalities, origin, arterial course, and any aberrations in the main vessel or its termination.^4^ Comparative analyses in the literature indicate that anomalous origin of the right coronary artery (RCA) is approximately six times more prevalent than that of the LCA, although it is associated with fewer adverse outcomes.^5^

Whenever an intervention is warranted, either due to symptoms or high-risk features (discussed later), surgical options such as unroofing or reimplantation are preferred over coronary artery bypass grafting to mitigate long-term graft-related complications, including occlusion.^6^

## Summary figure

### Timeline

**Table:** 

	
Day 0	Attended cardiology clinic for chest pain assessment and referred for further investigation.
Week 2	Computed tomography coronary angiogram (CTCA) was performed that showed anomalous right coronary artery (RCA) insertion.
Week 3	Admitted due to the above and reassessed on the ward. She was then referred for exercise stress testing that revealed inducible inferior territorial ischaemia on electrocardiogram (ECG) and echocardiogram (ECHO).
Week 4	The patient reported no further symptoms as reviewed in the outpatient clinic. She was further discussed at multidisciplinary meeting (MDM) involving cardiologists, geneticists, cardiac surgeons, and Inherited cardiac conditions (ICCs) team, and a decision was made for her to be seen at the tertiary centre.
Week 10	The patient was reviewed at the tertiary center and plan made regarding treatment options. The patient reported no further disabling symptoms. Further illustration is presented in the case report.

## Case presentation

We present the case of a 35-year-old female patient who was referred to our cardiology outpatient clinic due to intermittent chest pain, experienced both on exertion and occasionally at rest. Over the preceding 4–6 months, chest pain had progressively become more exertional, typically manifesting during physical activity such as playing with her children and resolving with rest. For a few year prior, she had been experiencing infrequent palpitations an episode of near syncope many years ago, but no abnormality was ever found on ambulatory electrocardiogram (ECG) monitoring. There was no recent history of syncope or heart failure symptoms.

Her medical history was significant for well-controlled dyslipidaemia and mild hypertension. Although there was no family history of sudden cardiac death, she reported a paternal family history of premature coronary artery disease (pCAD). She was a nonsmoker and reported occasional alcohol consumption. Her medications included atorvastatin 40 mg nocte, bisoprolol 2.5 mg once daily for palpitations, perindopril 2 mg once daily, and glyceryl trinitrate (GTN) sublingual spray as needed prescribed by her general practitioner (GP) prior to referral. Her calculated Q-RISK3 was 1.5%.

Cardiovascular examination was unremarkable. Blood pressure measurements were within normal limits bilaterally (left arm: 122/60 mmHg and right arm: 118/64 mmHg), and the remainder of the physical examination was normal. A 12-lead ECG demonstrated normal sinus rhythm with subtle horizontal ST-T segment sagging in lead II and III (see *Figure 1* below).

**Figure 1:**
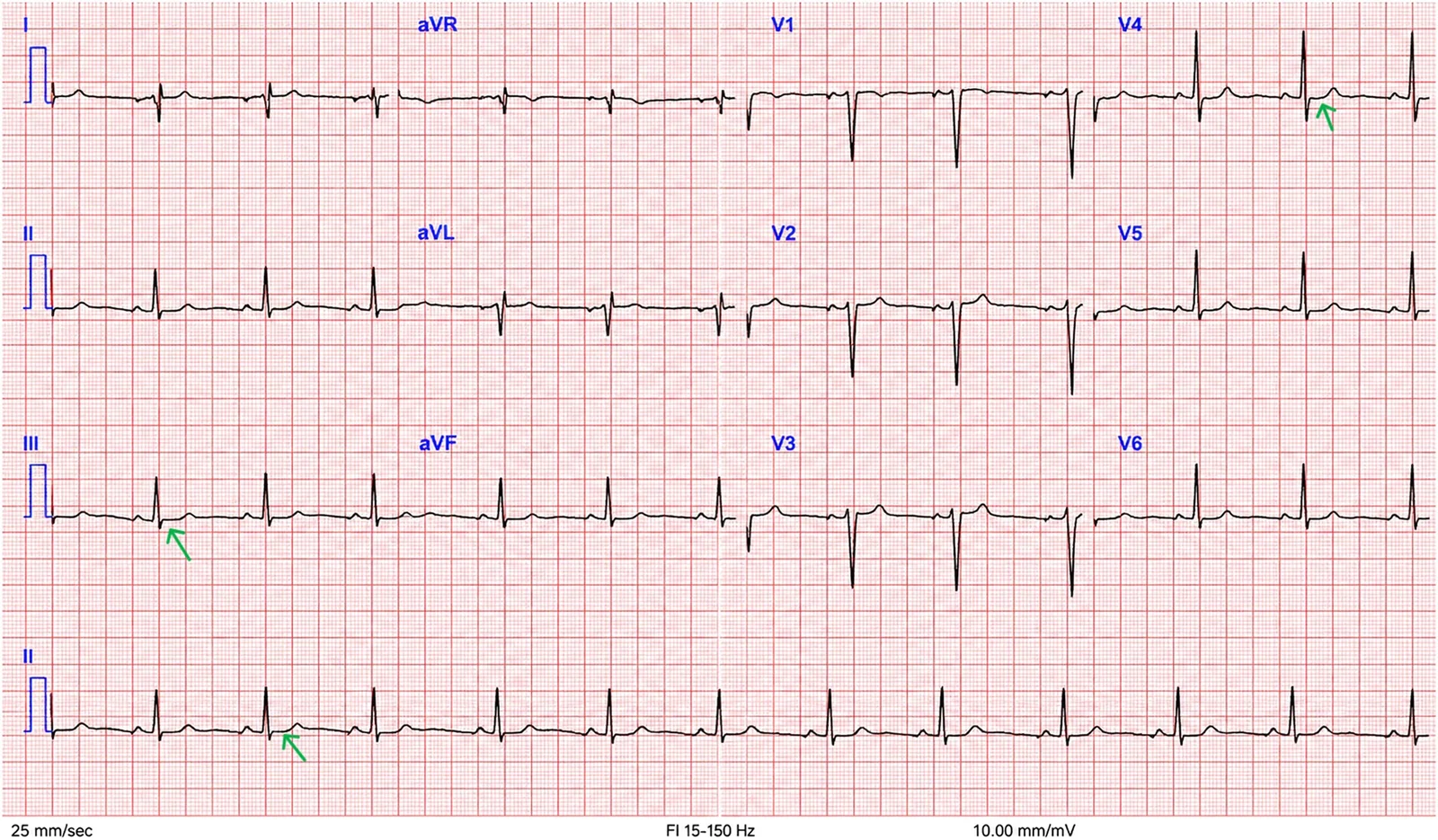
12-lead ECG showing subtle ST-T sagging in lead V4, II & III (marked by arrows).

Her blood investigation in outpatient clinic at initial contact revealed a mildly raised total cholesterol level of 5.4 mmol/L (recommended range < 5 mmol/L); LDL-Cholesterol 3.28 mmol/L (normal < 3 mmol/L). However, the rest of the results, including haemoglobin A1c, brain natriuretic peptide, renal profile, and thyroid function, was normal.

Given her history of intermittent exertional chest pain, hypertension, mild dyslipidaemia, and family history of premature CAD, further diagnostic evaluation was undertaken. Computed tomography coronary angiography (CTCA) and transthoracic echocardiography (TTE) were performed. The echocardiogram demonstrated a structurally normal heart with a left ventricular ejection fraction of 55%.

In contrast, CTCA revealed an anomalous origin of the RCA arising from the left coronary sinus. The artery followed an inter-arterial course, considered ‘malignant’, passing between the aorta and pulmonary artery. This anatomical configuration resulted in compression of the proximal one-third of the vessel, extending to the segment proximal to its major branches (see *Figures 2–5*).

**Figure 2:**
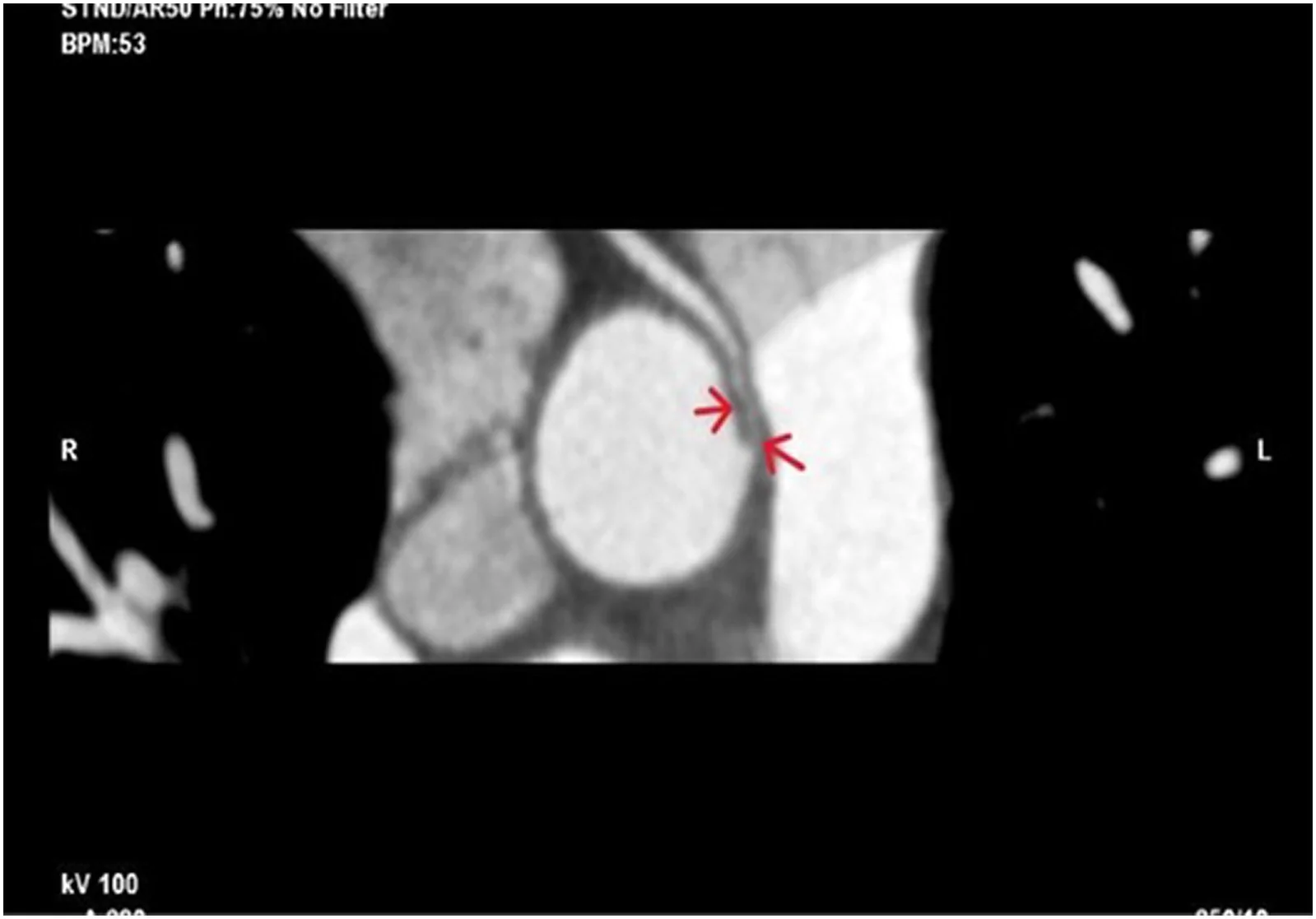
CT coronary angiogram sagittal section—arrows depict malignant inter-atrial right coronary artery (RCA) course originating from the left coronary cusp (LCC)—magnified image.

**Figures 3:**
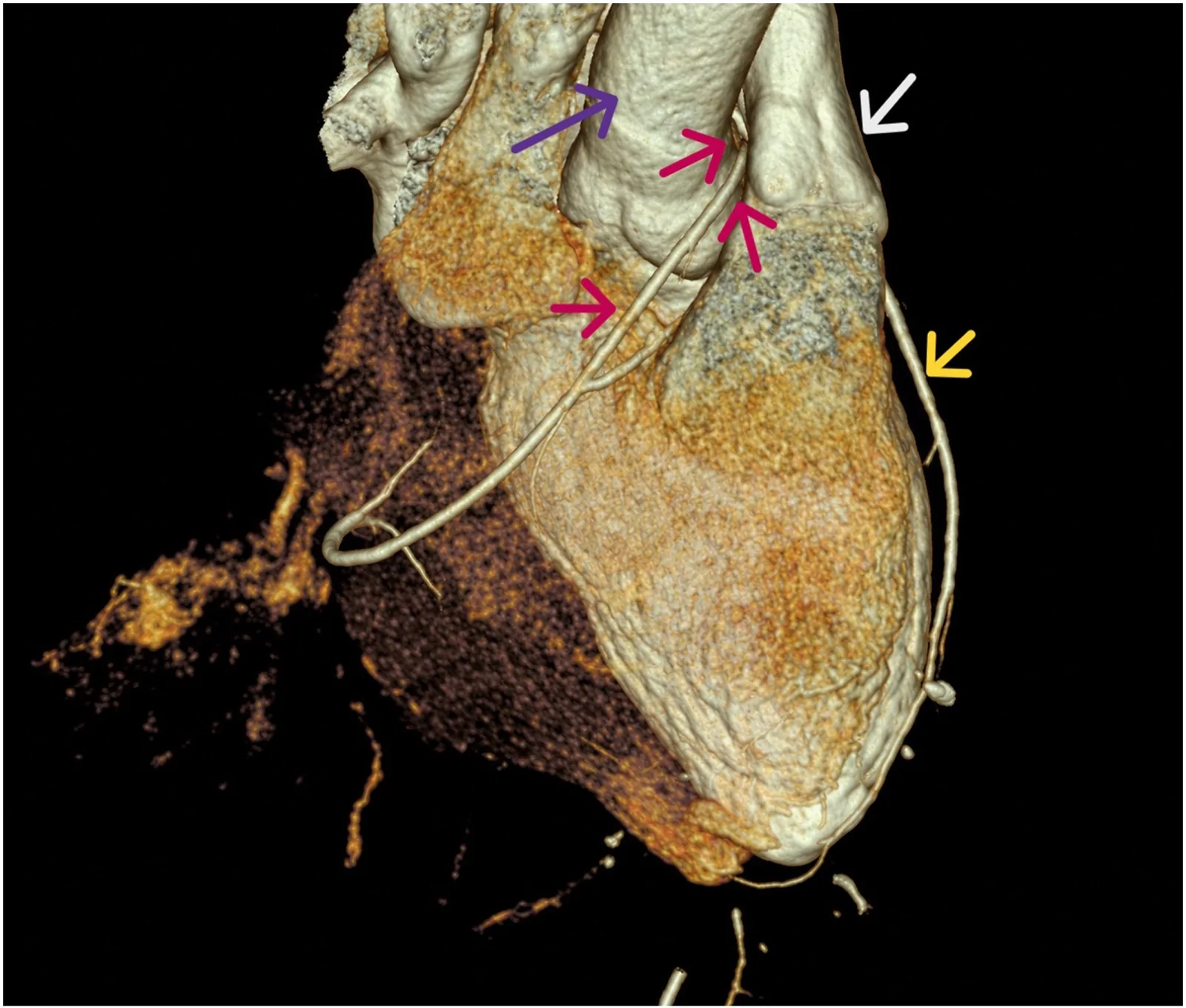
3D volume rendered image on CTCA showing anomalous inter-arterial (malignant) origin of RCA from left coronary sinus. Red arrows show malignant inter-arterial course of the RCA, yellow arrow depicts the Left anterior descending artery (LAD), purple arrow shows the Aorta/Aortic root and white arrow: pulmonary artery. Gated CT Image in 43 RAO 28 CRA (**RAO 43°** = **43°** Right Anterior Oblique viewing angle, **CRA 28°** = 28°Cranial angulation).

**Figure 4:**
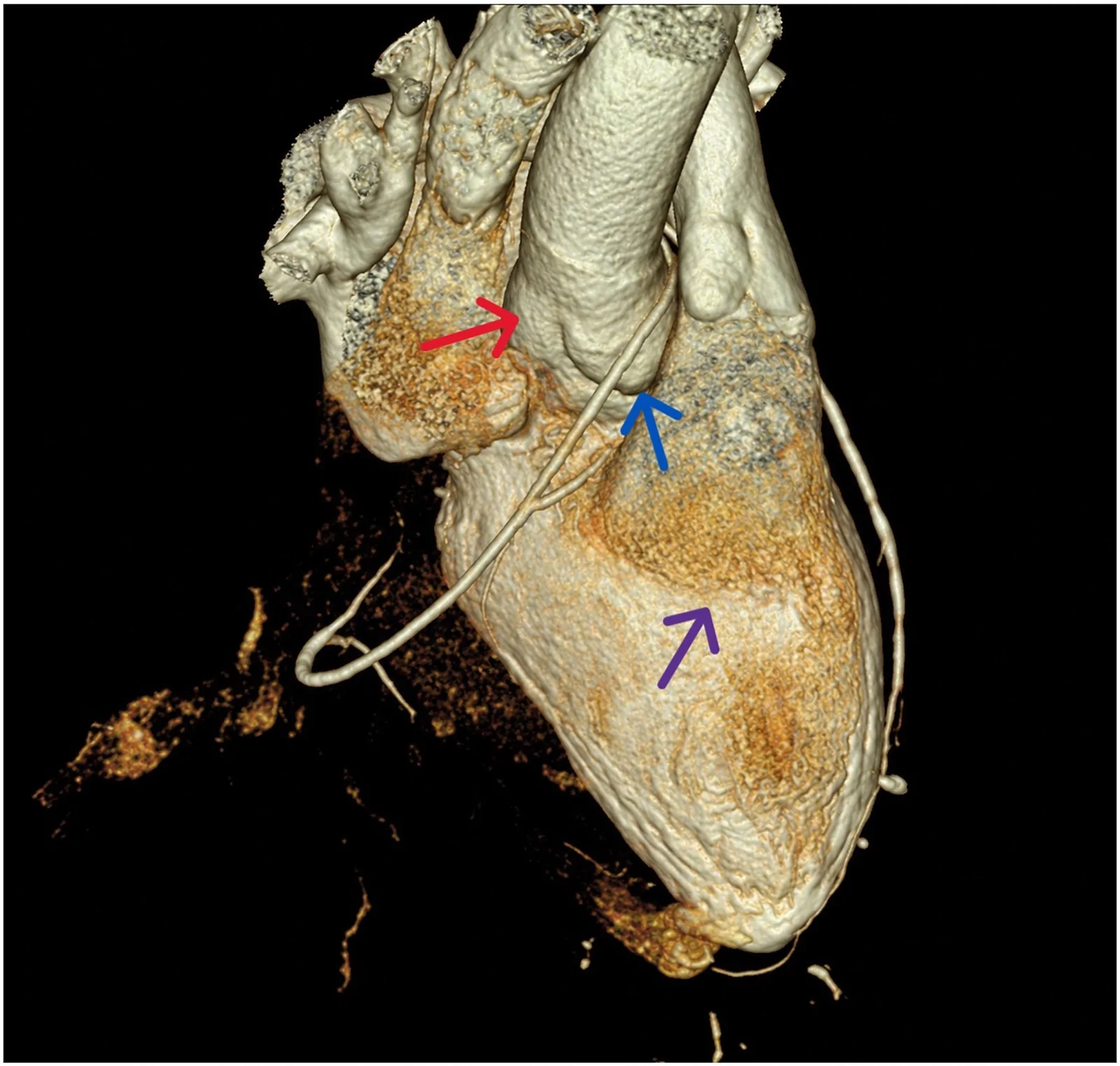
3D volume rendered image on CTCA in 41^0^ RAO 29^0^ cranial view. Red arrow: Left coronary cusp (LCC) with both RCA and LCA originating anteriorly; blue arrow pointing towards Right coronary cusp (RCC); purple arrow pointing at Right ventricle (RV) towards the RVOT (RV outflow tract).

**Figure 5:**
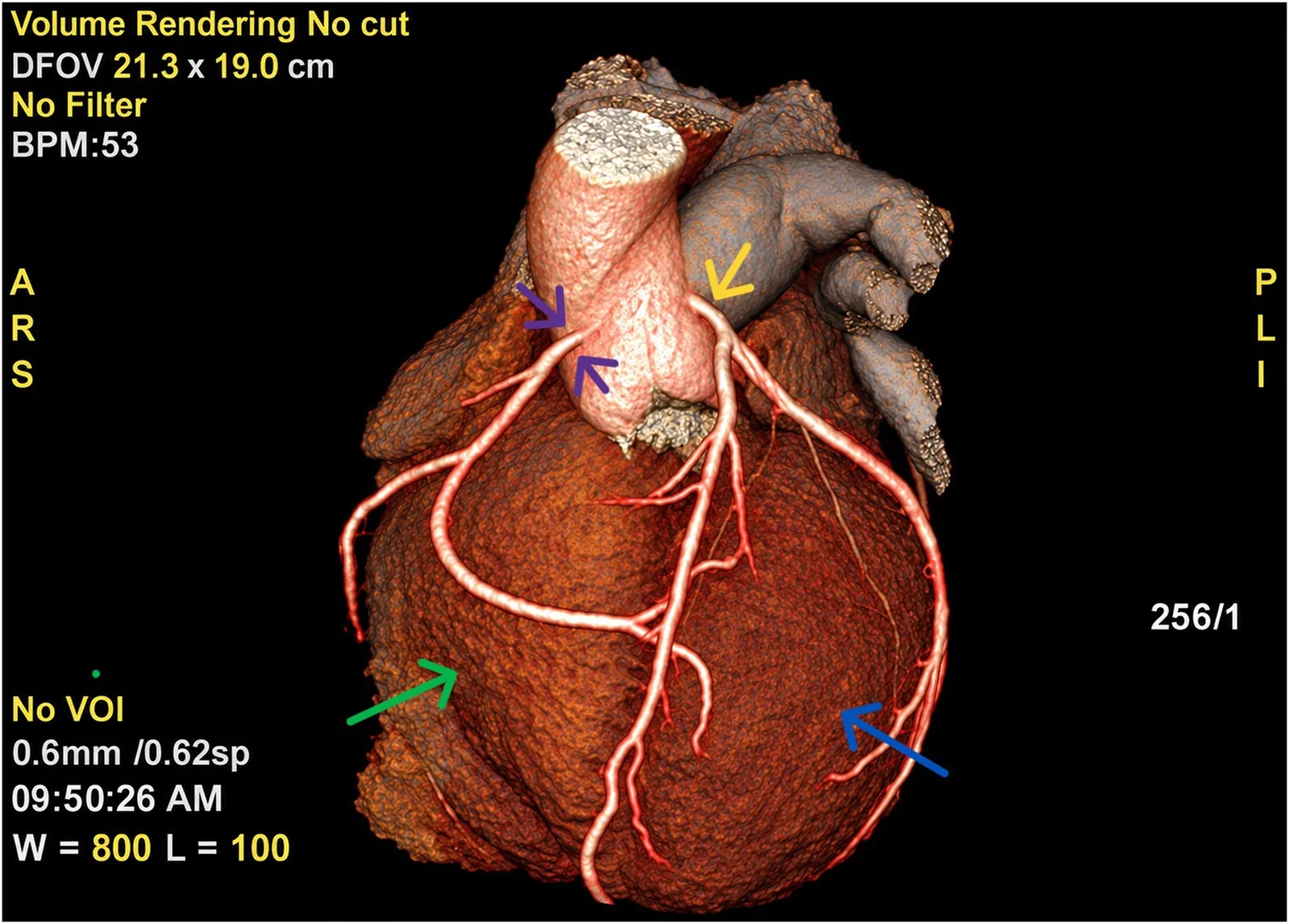
3D volume rendered image on CTCA in 57**°** LAO 33**°**CRA (left anterior oblique, cranial view); dark purple arrow depicts anomalous origin of RCC from LCC; yellow arrow depicts left main coronary artery bifurcating into LAD and left circumflex artery; blue arrow depicts left ventricle; green arrow depicts the right ventricle (RV).

No evidence of atherosclerotic plaque was identified in any of the coronary arteries. Given the diagnosis of a malignant course of the right coronary artery, she was admitted for further evaluation to the coronary care unit and her case reviewed at the cardiology multidisciplinary team (MDT) meeting.

For further evaluation, the patient underwent an exercise stress echocardiogram with simultaneous ECG monitoring. At rest, all myocardial segments demonstrated preserved viability and normokinesia with maintained myocardial thickness. At peak exercise, the patient did not report chest pain; however, ECG findings indicated mild ischaemia in the inferior septum with possible mild ischaemia in the basal inferior segments.

The corresponding ECG revealed horizontal to upsloping ST-segment depression accompanied by T-wave inversions in the inferior and lateral leads, consistent with ischaemia in the RCA territory; she, however, remained asymptomatic (see *Figure 6* below).

**Figure 6:**
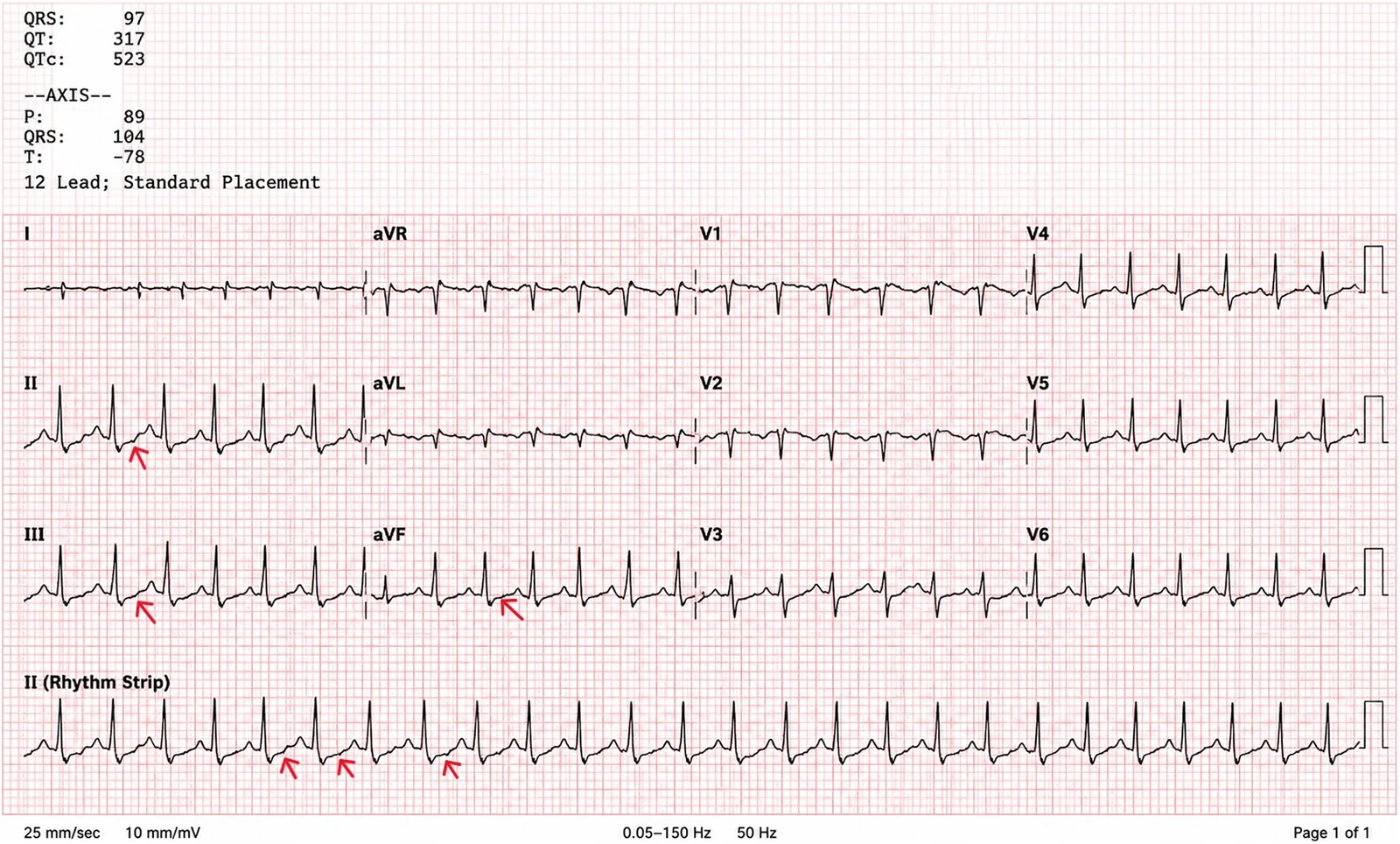
Exercise stress test; ECG at peak stress showing horizontal-to-upsloping ST depressions in inferolateral leads marked by red arrows in II, III, aVF.

## Outcome and follow-up

The results were discussed with the patient, who was advised to continue her current medical therapy, including Aspirin 75 mg daily, atorvastatin 80 mg once daily, GTN as well as her antihypertensive medication al mainly for primary prevention of an ischaemic coronary event. She was referred to a regional adult congenital heart disease centre for specialized management and will continue follow-up there, with consideration of intervention on the anomalous RCA, if clinically indicated.

At the time of referral, no decision was made to proceed with surgical correction following MDT discussion, given the absence of consistent physical symptoms on her exercise stress test despite subtle ischaemic changes on echocardiography and ECG.

Surgical intervention was considered to carry a higher risk of morbidity compared with conservative medical management. She will be reviewed again in 1 year, at which time intervention will be reconsidered if the potential benefits of correction clearly outweigh the periprocedural risks and associated post-op morbidity, as advised by the surgical team.

## Discussion

At least 50% of patients with anomalous coronary artery insertion present with exertional angina, now termed chronic coronary syndrome.^7^ Most cases are due to atherosclerotic epicardial CAD, caused by a mismatch between myocardial oxygen demand and supply during exertion. This requires a high index of clinical suspicion to avoid underdiagnosis in younger patients, especially in those with typical symptoms. One such entity is an anomalous origin of the coronary arteries, most commonly the RCA arising from the left coronary sinus.^8^

According to an extensive literature review, the incidence and clinically meaningful classification of aberrant RCA were first described in 1969 and further refined in 2000.^9^ The need to classify these lesions is emphasized in the literature by the fact that, in any individual, an inter-arterial course of a coronary artery might be associated with a higher risk of major adverse cardiovascular events.^10^

Currently, CTCA is the gold-standard investigation with cardiac magnetic resonance imaging studied as an alternative to CTCA with added benefit of assessing the functional significance an anomalous artery could have on coronary blood flow and tissue characterization to establish a cause–effect relationship.^11^

Invasive coronary angiography, once the definitive diagnostic tool and later superseded by CTCA, has seen resurgence in application when combined with intravascular ultrasound and, to a lesser extent, optical coherence tomography to assess vessel geometry, stenosis severity, and suitability for intervention.^12^

TTE and transoesophageal echocardiography also play an important noninvasive role, particularly in children, allowing visualization of coronary origins, ostia, and sometimes the proximal vessel. The 2021 European Society of Cardiology (ESC) Adult Congenital Heart Disease guidelines recommend provocative testing when an anomalous coronary artery is incidentally detected or diagnostically uncertain, with exercise stress testing as the preferred initial modality.^13^

Systolic compression from aorta rather than pulmonary artery in the case of interarterial course, seems more plausible as a cause driving ischaemia in such cases. Angelini *et al.* postulated an alternative to the conventional thought process that some part of the aberrant artery (inter-arterial) may have altered characteristics as it runs intramurally for the initial part that could promote ischaemia.^14^  *Benjanuwattra et al.*, in one of their reviews, commented on surgical options for anomalous RCA. They found that in difficult situations where unroofing and reimplantation was not possible, coronary artery bypass surgery could be tried albeit surgeons still concerned about risk of graft-failure likely secondary to competitive flow.^15^

An anomalous origin of the RCA from the left coronary sinus is very rare; therefore, the choice between continued medical therapy and surgical treatment relies on clinical judgement of MDTs.

## Lead author biography


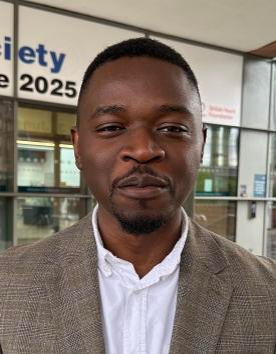


Cardiology ST6 registrar, currently in my third year of core cardiology training in Leeds General Infirmary, UK. I have a special interest in heart rhythm disturbances and inherited cardiac conditions.

## Data Availability

The data underlying this article are available in the article and in its online supplementary material.
